# A path analysis on the direct and indirect effects of the unit environment on eating dependence among cognitively impaired nursing home residents

**DOI:** 10.1186/s12913-019-4667-z

**Published:** 2019-10-30

**Authors:** Alvisa Palese, Luca Grassetti, Valentina Bressan, Alessandro Decaro, Tea Kasa, Melania Longobardi, Mark Hayter, Roger Watson

**Affiliations:** 1 Department of Medical Sciences, Udine University, Viale Ungheria, 20, 33100 Udine, Italy; 2 Department of Economics and Statistics, Udine University, Via Francesco Tomadini, 30, 33100 Udine, Italy; 3 Faculty of Health Sciences, Hull University, Hull, HU6 7RX UK

**Keywords:** Eating dependence, Feeding difficulties, Mealtime difficulties, Nursing home, Path analysis, Dementia, Cognitive decline

## Abstract

**Background:**

This study aims to estimate the direct and indirect effects of the unit environment alongside individual and nursing care variables on eating dependence among residents who are cognitively impaired and living in a nursing home.

**Method:**

A multicentre observational study was carried out in 2017: 13 Italian nursing homes were involved in data collection. Included residents were aged > 65 at baseline, living in the considered facility for the last 6 months and during the entire study period and having received at least one comprehensive assessment. Data were collected (a) at the individual level: eating dependence using the Edinburgh Feeding Evaluation in Dementia Scale and other clinical variables; (b) at the nursing care level with daily interventions to maintain eating independence assessed with a checklist; and (c) at the nursing home level, using the Therapeutic Environment Screening Survey for Nursing Homes.

**Results:**

One thousand twenty-seven residents were included with an average age of 85.32 years old (95% CI: 84.74–85.89), mainly female (781; 76%). The path analysis explained the 57.7% variance in eating dependence. Factors preventing eating dependence were: (a) at the individual level, increased functional dependence measured with the Barthel Index (β − 2.374); eating in the dining room surrounded by residents (β − 1.802) as compared to eating alone in bed; and having a close relationship with family relatives (β − 0.854), (b) at the nursing care level, the increased number of interventions aimed at promoting independence (β − 0.524); and (c) at the NH level, high scores in ‘Space setting’ (β − 4.446), ‘Safety’ (β − 3.053), ‘Lighting’ (β − 2.848) and ‘Outdoor access’ (β − 1.225). However, environmental factors at the unit level were found to have also indirect effects by influencing the degree of functional dependence, the occurrence of night restlessness and the number of daily interventions performed by the nursing staff.

**Conclusion:**

Eating dependence is a complex phenomenon requiring interventions targeting individual, nursing care, and environmental levels. The NH environment had the largest direct and indirect effect on residents’ eating dependence, thus suggesting that at this level appropriate interventions should be designed and implemented.

## Background

The progressive decline of functional dependence has been reported as the major cause of nursing home (NH) admission among cognitively impaired older individuals [1]. In the moderate stage of dementia, by following a hierarchical order, impairments among early-, middle- and late- loss activity of daily living (ADL) have been documented, with eating independence being among the last ADLs to deteriorate [2]. Initially, residents have been documented to refuse to eat [3], and then to manifest behavioural disorders, dysfunctions of eating mechanism, the inability to recognise food and how to use cutlery; in the late stages, complex compensatory and supportive measures during mealtime are required [4].

Aiming at preventing negative consequences such as malnutrition, anorexia, increased occurrence of pressure ulcers, dehydration, aspiration, multiple hospitalisation and mortality [5, 6], several interventions both at the resident and at the environmental levels [7] have been documented to date. Among the first, simple (e.g., offering verbal prompts, modified food and drinks and finger food [8]) to complex interventions (e.g., space retrieval, Montessori method [9]) have been studied. Moreover, the effectiveness of training programs aimed at educating caregivers and healthcare professionals on safe methods capable of guaranteeing optimal eating assistance [10, 11] has also been researched. At the environmental level, homelike dining rooms with limited noises or distractions during mealtime; appropriate meal service delivery styles and soft music during mealtime, have been documented to increase intake [10, 11]. However, these studies have been confined in the environment where meals are eaten [12]. Instead, residents live in complex NH environments for several years, not spending all day in the dining room where these changes have been recommended to be implemented.

According to Lin et al. [9] residents spend from 11.25 to 19.03 min to complete their meal; therefore, they are immersed in the dining environment for around 1 h and half a day, suggesting that they are more exposed to the influence of the entire physical environment of the NH unit that requires changes in their design choices [13].

However, to date only a few studies have been performed to identify the role of the NH environment alongside other individual and nursing care factors in initiating or in delaying the onset of disability in eating [14, 15]. Moreover, according to our best knowledge, no studies have investigated the possible direct and indirect effects of the NH environment on the degree of dependence in eating [14], for example influencing other variables directly affecting eating performance. Furthermore, given that eating is a social process [16], whether residents are used to eating at a table with other residents or alone in their bedroom has not been considered in available studies.

Therefore, the main aim of this study was to estimate the direct and indirect effects of the NH unit environment alongside individual and nursing care variables on residents’ eating dependence. We hypothesised that beyond the role of individual and nursing care factors [12] on the degree of dependence in eating, other environmental factors at the NH unit level, directly and indirectly increase and/or prevent eating impairments.

## Methods

### Study design

A multicentre pragmatic observational study design was performed in 2017, and here reported according to the STrengthening the Reporting of OBservational studies in Epidemiology studies [17].

### Sample and setting

A total of 13 public NHs located in a rural area in the North-East of Italy where around 100,000 citizens were living at the time of the study and under the same Regional Health Service rules were preliminary assessed for their homogeneity in (a) their mission as long term facilities, (b) the amount of nursing care offered daily by nursing aides (NAs) and Registered Nurses (RNs) in around 75 min/day/resident, and (c) the admission resident criteria, as residents with moderate/severe functional dependence due to different health conditions - mainly dementia [18]. All NH were approached and all agreed to participate; these were equipped with an average of 86 beds (from 33 to 200, a total of 1161) and at the period of the study were hosting on average 83 residents (from 30 to 164).

**Fig. 1:**
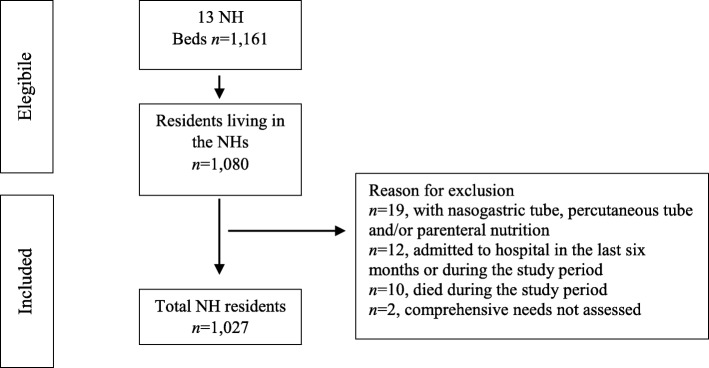
Eligible residents, included and reasons for exclusion. *NH* Nursing Home, *n* number

Residents who were (a) > 65 years; (b) living in the same NH unit for the last 6 months and during the entire study period; and (c) who had reported in his/her records a comprehensive need assessment were deemed eligible. In Fig. 1, the flow diagram of residents included in the study has been reported.

### Variables and data collection process

The outcome variable of the study was the eating performance as measured using the Edinburgh Feeding Evaluation in Dementia scale (EdFED) [19] in its Italian validated version [20]. The tool consists of 10 items based upon a 3-point Likert scale (0 never, 1 sometimes, 2 often). It was completed by observing each resident during their entire mealtime.

The tool has demonstrated strong psychometric properties in previous studies [e.g., 19, 20]. As the total score increases (from 0 to 20), the dependence in eating is higher. The item number 11, measuring the appropriate level of care required by the resident (only supportive/educative; partly or wholly compensatory), was also filled in by giving a score of 0, 1 or 2, respectively, according to the care delivered during the observed mealtimes.

The remaining variables were collected at the following levels, as reported in Table 1:

1. Individual level: in addition to some demographic variables (age, gender), the degree of functional dependence (Barthel Index, [21]), the cognitive performance (Cognitive Performance Scale, [22], the emotional status (Depression Rating Scale, [23]), the presence of pain and its intensity (Pain Intensity, [24]), the occurrence of some behavioural symptoms (night restlessness, verbal aggressiveness, physical aggressiveness occurrence) and the clinical instability (Clinical Instability Score, [25]) were considered. The presence of close/intimate relationships with family relatives [25] and where the resident was used to have breakfast, lunch and dinner (in his/her bedroom or in the dining room), and with whom (alone or with others) was also considered.
2. Nursing care level: routine interventions performed to maintain eating independence at the (a) resident level (e.g., verbal, behavioural or motivational prompts) and at the (b) environmental level (e.g., by promoting the desire to eat by stimulating smell and visual memory, creating and maintaining a peaceful environment allowing residents’ concentration) were recorded. These interventions emerged in a parallel study performed in the same NHs through focus groups [18] and then transformed into a checklist in the current study as a basis for observation.
3. Nursing Home level: in addition to the size (number of beds), the organisation of the NH in the number of units as a confined environment (with no commons areas to serve more than one unit) where a group of residents were living at the time of the study, cared for by a nursing team led by a nurse leader (26), was assessed. Therefore, the therapeutic properties of each NH unit environment were evaluated using the Therapeutic Environment Screening Survey for Nursing Home (TESS-NH) [26] composed of 84 items categorised into 13 domains as reported in Table 1. Authorisation to use the tool was obtained from the author (Prof. Sloane, correspondence available from authors). After having ensured the cross-cultural and conceptual equivalence, together with the face and content validation, the tool was subjected to the validation of other properties: as according to the Authors the tool is a checklist, only the inter-rater reliability, the test−retest, the criterion validity, the inter-dimension correlations and the internal consistency were evaluated (available from authors).

**Table 1:** Variables measured, metrics and validity of the measure, and source(s) of data collection

Outcome	Measure metrics and validity evidence documented in available studies [in brackets] and as emerged in this study	Source(s) of data
Edinburgh Feeding Evaluation in Dementia scale (EdFED)	From 0 total independent, to 20 totally dependent [19, 20] Reliability Cronbach alpha = 0.909	Direct observation during lunch time
Appropriate level of care required by the resident (only supportive/educative; partly or wholly compensatory) (EdFED)	From 0 to 2 [19, 20]	
Individual level		
Age, Sex	–	Data was extracted from the NH and regional database
Functional dependence using the Barthel Index (10 items)	From 0 total dependent, to 100 total independent [21] Reliability Cronbach alpha = 0.945	
Cognitive Performance Scale (6 items)	From 0 intact, to 6 severe impairment; scores ≥4 indicate moderate/severe impairment [22] Reliability Cronbach alpha = 0.937	
Depression Rating Scale (14 items)	From 0 to 14; scores ≥3 indicate minor or major depressive disorders [23] Reliability Cronbach alpha = 0.834	
Pain Intensity Scale (1 item)	From 0 no pain, to 3 severe pain [24]	
Night restlessness (1 item)	Each scored from 0 absent, to 4 always present [25]	
Verbal aggressiveness (1 item)		
Physical aggressiveness (1 item)		
Clinical Instability Score (1 item)	From 0 clinically stable, to 4 high instability [25]	
Close/intimate relationships with family relatives (1 item)	Yes/no [25]	
Where the resident habitually eats -in his/her bedroom, alone -in the dining room, near one resident (on left or right side) -near two residents (on left and right) -near two residents (on left/right and in front) -surrounded by other residents (on left, right and in front)	From 0 eating alone, to 4 surrounded by other residents	
Nursing care level		
(a) environmental interventions: starting the mealtime ritual by stimulating hearing and sight memory (1. ringing a bell, 2. opening the dining room), promoting the desire to eat by stimulating smell and visual memory (3. setting the tables in advance as in a restaurant, 4. entering the dining room with a meal trolley); creating and maintaining a peaceful environment allowing residents’ concentration (5. lowering distracting stimuli; 6. balancing the presence of the family)	(a) From 0 none, to 6 all interventions are daily performed [18]	Nursing Staff observation via checklist
(b) resident interventions: knowing the resident (1. collecting and sharing their stories and habits; 2. understanding their daily variances and adapting routines; 3. establishing residual self-feeding abilities), escalating feeding care (4. verbal, 5. behavioural, 6. motivational prompts; 7. respecting refusals and waiting; 8. balancing insistence and resistance; 9. deciding the best position), de-escalating difficulties due to meals/utensils (10. by adapting the consistency of food, and utensils)	(b) From 0 none, to 10 all interventions are daily performed [18]	
NH level		
Units composing each NH Beds of the NH unit where each resident was living	Number Number	Data was extracted from the regional database
Therapeutic Environment Screening Survey for Nursing Home (TESS-NH) (84 items categorised in 13 domains [26, 27]	Higher scores in each domain indicates high environment quality [26, 28] Reliability resulted in all evaluable dimensions in Cronbach alpha = > 0.600	Direct observation
- ‘Unit autonomy’: nursing station presence/type; nursing station for paperwork; desk for paperwork; combined work area for paper work; enclosed workroom, not a nursing station; unit use as pathway between other units; residents eat on/off units; formal activities on/off unit; residents bathe on/off unit (9 items)	- Scores 0, 1, 2 or 3 according to the item	
- ‘Outdoor access’: enclosed courtyard; attractiveness of courtyard; courtyard is functional (3 items)	- Scores 0, 1, 2 or 3 according to the item	
- ‘Privacy’: privacy curtain provides only separation between beds in semiprivate rooms (1 item)	- Score 0 or 1	
- ‘Exit control’: doors of rest of facility distinguished; doors to outside distinguished; number of exits off of the unit; number of elevators off of the unit; doors are looked; locking device triggered by approach; look disengaged by keypad/switch; looked at night/during bed weather; doors are alarmed; alarm triggered by device worn by resident; alarm disengaged using keypad, card or switch (12 items)	- Scores 0, 1 or 2 or Not applicable according to the item -	
- ‘Maintenance’: maintenance of social space; of halls; of residents’ rooms; of resident bathrooms (4 items)	- Scores 0, 1 or 2	
- ‘Cleanliness’: cleanliness of social spaces; of halls; resident rooms; resident bathrooms; bodily excretion odour in public area; in resident rooms (6 items)	- Scores 0, 1 or 2	
- ‘Safety’: floor surfaces in social spaces; in halls; in resident rooms; in resident bathrooms; handrails in hallways; in bathrooms (6 items)	- Scores 0, 1 or 2	
- ‘Lighting’: intensity in hallways; in activity areas; in resident rooms; glare in hallways; in activity areas; in resident rooms; lighting evenness in hallways; in activity areas; in resident rooms (9 items)	- Scores 0, 1, 2 or 3 according to the item	
- ‘Visual/tactile stimulation’: bedrooms with a view of the courtyard; public areas with a view of the courtyard; tactile stimulation opportunities; visual stimulation opportunities (4 items)	- Scores 0, 1 or 3	
- ‘Noise’: status of the television in main activity areas; resident screaming/calling out; staff screaming/calling out; T/radio noise; loud speakers/intercom noise; alarm/call bell noise; other machine noise (7 items)	- Scores 0, 1, 2 or 6	
- ‘Space/seating’: % of rooms with a chair per person; public room inventory; path leads to dead ends; path with places to sit; configuration of rooms on unit (5 items)	- From 0 to 1, 2 or 3 or Not applicable according to the item	
- ‘Familiarity/home likeness’: public areas homelike; kitchen of the unit; pictures/mementos in resident room; non-institutional furniture in resident rooms; residents’ appearance (5 items)	- Scores 0, 1, 2 or 3 according to the item	
- ‘Orientation/cueing’: doors left open; resident’s name on/near door; current picture of the resident; old picture of the resident; object of personal significance; room numbers; colour coding; bathroom door left open, toilet visible from the bed; bathroom door left open; toilet non visible from the bed; bathroom door closed, picture or graphic; activity areas visible from the 50% of resident rooms; visual indicator of activity areas visible from 50% of resident rooms; direction, identification sign visible from 50% of resident room (13 items)	- Scores 0 or 1	
Therapeutic Environment Screening Survey for Nursing Home (TESS-NH) global single item (1 item)	From 1 low, distinctly unpleasant, negative, non-functional, to 10 high, quite pleasant, positive, and functional environment [26]	Direct observation

*ADL* Activity of Daily Living, *NH* Nursing Home, *RN* Registered Nurse, *TESS-NH* Therapeutic Environment Screening Survey for Nursing Home

Path analysis findings: direct

### Data collection process

Facilities were approached by the research team in the second semester of 2017. Different methods of data collection were used according to the level and nature of the data. In larger NHs data were collected over a few days:

- Nursing Home level: two trained researchers with a nursing background visited each NH unit, taking around 2 h to complete the data collection with the TESS-NH [26]. Data were collected independently and then agreed upon, and discordances were discussed with a third researcher. Residents who were living in each NH unit on the day of the survey (*n* = 1080) were registered as eligible to be included in the study.
- Outcome variable and individual level variables: after 3–4 weeks, on a day selected randomly by the principal investigator, the outcome was measured by observing each resident during lunch time in the dining room or at his/her bedside according to his/her routines. Four researchers with a nursing background and trained via a 4-h course in the use of the Edinburgh Feeding Evaluation in Dementia scale [19, 20] were involved. Only residents satisfying the inclusion criteria (*n* = 1027) were evaluated. For these residents the last complete comprehensive assessment performed and recorded in the NH and regional databases with the Val. Graf tool [25] including different measures was extracted after having received the appropriate authorization from the Ethical Committee.
- Nursing care level variables: the daily interventions at the environment and at the individual level performed by the staff in the dining room or in the bedroom to maintain eating performance [18] were observed on the day of data collection at the individual level trough observations based on a checklist [18].

### Bias control

At the resident level, selection bias [29] was prevented by including all residents living in the approached NHs. To avoid any misclassification [29] validated tools were used [19–23, 26]; moreover, their reliability was reassessed with the data collected in this study as reported in Table 1. Furthermore, while the comprehensive need assessment was performed by trained RNs, responsible for the care delivered to the residents, the outcome variable and the nursing care variables were assessed by researchers not involved in the daily care of residents after having received appropriate training and under the supervision of an expert researcher.

At the NH level, performance bias was prevented by including residents who were receiving the same amount of nursing care as established by regional rules. During the study period the NH policies were stable over time. Furthermore, to prevent any bias in observation, the NH unit environment evaluation with the TESS-NH tool [26] and by the same trained researchers not involved in other data collection and in daily care of residents.

### Modelling and data analysis

On a preliminary fashion, Descriptive and inferential statistics were performed by computing frequencies, percentages and averages (with Standard Deviations [SD], ranges; or Confidence Intervals [CI] at 95%).

Then, in line with the study hypothesis, the Intra Class Correlation (ICC) was evaluated under random and fixed effects (CI at 95%, bootstrap method) to identify effect clusters at the NH units’ levels on the outcome variable. The ICC of the Edinburgh Feeding Evaluation in Dementia scale scores (19) at the NH level were 0.10 (95% CI: 0.03–0.19) and 0.06 (95% CI: 0.02–0.15) under random and fixed effects, respectively; at the NH units, these were 0.13 (95% CI: 0.06–0.20) and 0.10 (95% CI: 0.07–0.20) under random and fixed effects, respectively.

Then, taking into account the high cluster effect of the NH unit on the outcome variable, the path analysis model was developed: on a preliminary fashion, all variables collected were introduced in the saturated model and tested. The complexity of the correlation structure forced a more sparing variable selection by including only those consistent with the available conceptual frameworks describing the relation between some (a) individual-, (b) NH-, and (c) nursing care- levels variables. Specifically, the Chang and Roberts model [30] was considered documenting that feeding difficulties are based on individual factors (memory and cognitive impairments), but also on several contingent factors that have a probabilistic relation with these difficulties attributable to time or space patterns. Among these, social and psychological factors, as well as the dining environment, and culturally appropriate food choices, have been identified. Thus, collected variables consistent with the above-mentioned model were explored in their correlations with the outcome variable (Additional file 1) and those significantly correlated with each other were kept. Some of those not significantly correlated were also included in the model according to the evidence available in the field (e.g., depression [31]; pain intensity [32]; verbal aggressiveness [33]; clinical instability [18]; and the environmental and resident interventions [7]). Collinearities were also then assessed and removed as in the case of the TESS-NH global item score and the TESS-NH dimensions. Finally, a direction of each relationship among variables was assigned according to the Chang and Roberts model [30] and the study hypotheses.

Thus, the path analysis was performed by introducing the outcome variable (=eating dependence) as measured with the Edinburgh Feeding Evaluation in Dementia scale [19, 20] and as explanatory variables those identified in the model: according to the study hypothesis, some variables at the NH level (e.g. bed size, and units) and at the individual level (e.g. age, gender, Cognitive Performance Score, clinical instability, close relationship with the family relatives’) [34] were considered as exogenous variables [35], not influenced by variables introduced in the model. The remaining were considered as endogenous variables [35].

Direct and indirect effects were then explored by sequential multiple regression analyses: the standardised coefficients β were estimated for each variable. Standard Errors (Std.Err), Test Statistics (z-values) and *p*-values (P(>|z|) were also reported to perform the inferential analysis. Moreover, according to Tarling [35] we considered (a) direct effects, (b) indirect effects (by simply multiplying the path coefficients connecting the causal variable to the outcomes) and (c) total effects (as the sum of direct and indirect effects). Therefore, the model fit was assessed analysing the coefficients of determination (*R*^2^) specific of each regression.

All analyses were performed by using the SPSS Statistical Package version 24 and R Core Team (R Core Team, 2017).

On a preliminary fashion, the database was checked for missing values (< 1%) and these were managed adopting the Full Information Maximum Likelihood approach [36]. The R Package Lavaan [37] was used for model estimation. The statistical significance was set at *p* < 0.05.

## Results

### Outcome variable

The 1027 residents included reported at the Edinburgh Feeding Evaluation in Dementia scale score on average 2.48 points (95% CI: 2.22–2.73); one fourth of them were in need of whole compensatory support (242; 23.6%) by carers as reported in Table 2.

**Table 2:** Outcome, individual, nursing care and NH level variables

Variables	N (%) averages (95% CI; SD)
Ouctome	
EdFED (0–20)	2.48 (2.22–2.73)
Level of care required, item 11 of EdFED	1.59 (0.84)
Supportive	659 (64.2)
Partial	126 (12.3)
Whole^a^	242 (23.6)
Individual level	
Age, years	85.32 (84.75–85.89)
Gender, sex	781 (76.0)
Barthel Index (0–100)	25.25 (23.47–27.03)
Cognitive Performance Scale (0–6)	3.35 (3.22–3.47)
Moderate/severely cognitively impaired (≥ 4)	471 (45.9)
Depression Rating Scale (0–14)	2.93 (2.72–3.13)
Minor or major depressive disorders (≥ 3)	459 (44.7)
Pain Intensity (0–3)	0.72 (0.66–0.77)
Night restlessness (0–4)	0.48 (0.42–0.55)
Verbal aggressiveness (0–4)	0.33 (0.28–0.38)
Physical aggressiveness (0–4)	0.14 (0.11–0.17)
Clinical Instability Score (0–4)	1.34 (1.29–1.40)
Close/intimate relationships with family relatives, weekly (yes)	710 (69.1)
Used to have meals	
In his/her bedroom, alone	223 (21.7)
Dining room, near one resident (on left or right side)	57 (5.6)
Dining room, near two residents (on left and right)	92 (9.0)
Dining room, near two residents (on left/right and in front)	97 (9.4)
Dining room, surrounded by other residents	558 (54.3)
Nursing care level	
Environmental Interventions (0–6)	3.76 (2.98–4.55)
Resident Interventions (0–10)	8.46 (7.39–9.52)
NH level	
Beds, number	92.6 (78.6–106.3)
Units in each NH, number	from 1 to 4
Beds at the NH unit level, number	34.38 (27.93–40.82)
TESS-NH dimensions	
Unit Autonomy (0–1.77)^b^	1.33 (0.00)
Outdoor Access (0–3)	2.10 (0.94)
Privacy (0–1)	0.35 (0.51)
Exit Control (0–1.20)	.91 (0.18)
Maintenance (0–2)	1.70 (0.36)
Cleanliness (0–2)	1.92 (0.16)
Safety (0–2)	1.84 (0.28)
Lighting (0–2.33)	1.85 (0.18)
Visual/Tactile stimulation (0–3)	2.42 (0.54)
Noise (0–2.5)^b^	1.76 (0.53)
Space Setting (0–1.75)^b^	1.80 (0.28)
Familiarity (0–2.60)^b^	0.84 (0.50)
Orientation/cueing (0–1)	0.38 (0.14)
TESS-NH global single item (0–10)	7.00 (1.55; range 5–10)

^a^whole compensatory care required

^b^as ranges, there were evaluated the average scores of each dimension [26]

*CI* Confidence of Interval, *EdFED* Edinburgh Feeding Evaluation in Dementia scale, *N* number, *NH* nursing home, *SD* Standard deviation, *TESS-NH* Therapeutic Environment Screening Survey for Nursing Home

### Individual, nursing care and NH level variables

As reported in Table 2, at the individual level, the residents’ age was on average 85.32 years (95% CI: 84.74–85.89) and the majority were female (781; 76%). The average score on the Barthel Index was 25.25 (95% CI: 23.47–27.03); nearly half of the residents were moderate/severely cognitively impaired (471; 45.9%) as reporting a Cognitive Performance Score ≥ 3 and, similarly, at the Depression Rating Scale, around half were diagnosed with minor or major depressive disorders (459; 44.7%). Considering the Pain Intensity Index, an average score of 0.72 out of 3 (95% CI: 0.66–0.77) was recorded, while in some specific behaviour problems such as night restlessness, verbal and physical aggressiveness, the average scores out of 4 were 0.48 (95% CI: 0.42–0.55), 0.33 (0.28–0.38) and 0.14 (0.11–0.17), respectively. The residents’ clinical instability was on average 1.34 out of 4 (95% CI: 1.29–1.40). The majority of them had a close relationship with their relatives (710; 69.1%) and they were used to have their meals in the dining room surrounded by other residents (558; 54.3%).

At the nursing care level, on a daily basis, an average of 3.76 out of six environment interventions (95% CI: 2.98–4.55) with a large range (from two interventions in seven NHs, to all six in four NHs) were performed. On a daily basis, at the resident level on average 8.46 out of 10 interventions (95% CI: 7.39–9.52) were performed with one NH performing only one and eight performing all interventions included in the checklist.

At the NH level, facilities were composed from one to 4 units with on average 34 residents. At the TESS-NH global score, the average rating was 7 out of 10 (SD, 1.55) ranging from 5 (six NH units) to 10 (3 units). Average scores in each TESS-NH dimension have been reported in Table 2.

### Path analysis

**Fig. 2:**
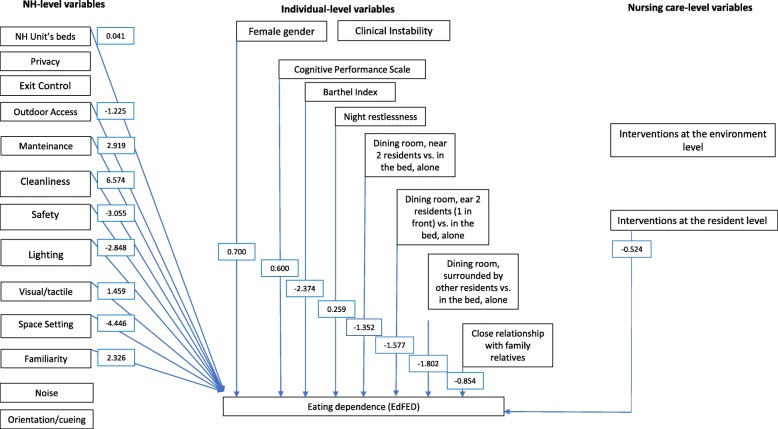
NH, individual and nursing care variables: direct effects on eating dependence as measured with the EdFED. In the boxes, β values are reported; the full specification of the model is reported in the Additional file 2. *EdFED* Edinburgh Feeding Evaluation in Dementia scale, *NH* Nursing Home

The model has explained the 57.7% variance in eating dependence as fully reported in the Additional file 2. As reported in Fig. 2, several individual level variables resulted as directly preventing self-feeding dependence. Higher scores on the Barthel Index had the greatest effect on preventing self-feeding dependence (β − 2.374). Compared to eating in bed alone, the following eating arrangements also resulted in preventing self-feeding dependence, although to a lesser extent: eating in the dining room with two residents (one on the left and one the right, β − 1.352), eating in the dining room with two residents (one of them in front, β − 1.577) or surrounded by other residents (β − 1.802) as compared to eating in bed, alone, have all emerged as preventing eating dependence. Moreover, having a close relationship with family relatives has also emerged as preventing eating dependence (β − 0.854).

At the nursing care level, the increased number of interventions performed on a daily basis at the resident level has emerged as a protective factor for eating dependence (β − 0.524). At the NH levels, with higher effects, ‘Space setting’ (β − 4.446), ‘Safety (β -3.055) ‘Lighting’ (β -2.848) and ‘Outdoor access’ (β − 1.225) dimensions have all resulted as preventing eating dependence.

On the other hand, factors directly increasing the likelihood of eating dependence at the individual levels were female gender (β 0.700), the increased Cognitive Performance Scores (β 0.600) and night restlessness (β 0.259) while the remaining variables entered in the model reported no significant contribution to the outcome variable. With the highest direct impact, some environmental dimensions as measured with the TESS-NH tool have increased the likelihood of eating dependence (‘Cleanliness’, β 6.574; ‘Maintenance’, β 2.919; ‘Familiarity’, β 2.326; and ‘Visual Tactile’, β 1.459). Also the unit’s bed size has increased the likelihood of eating dependence but with a limited direct impact (β 0.041).

**Fig. 3:**
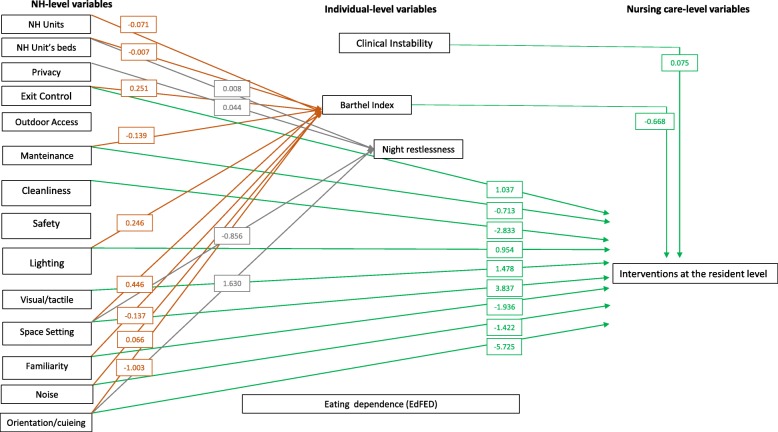
NH, individual and nursing care variables: indirect effects on eating dependence as measured with the EdFED. In the boxes, β values are reported; the full specification of the model is reported in the Additional file 2. *EdFED* Edinburgh Feeding Evaluation in Dementia scale, *NH* Nursing Home

As evident in Fig. 3, indirect effects have emerged mainly at the NH levels as measured with the TESS-NH. Functional dependence as measured with the Barthel Index [19] was prevented by the ‘Orientation/cueing’ (β − 1.003), ‘Maintenance’ (β − 0.139) ‘Familiarity’ (β − 0.137) dimensions, the number of units in the NH and the number of beds at the unit level (β − 0.071 and − 0.007, respectively).

In contrast, eating dependence was increased by ‘Space setting’ (β 0.446), ‘Exit control’ (β 0.251), ‘Lighting’ (β 0.246) and ‘Noise’ (β 0.006) dimensions.

Night restlessness was prevented by the ‘Space setting’ (β − 0.856), and increased by the ‘Orientation/cueing’ dimension (β 1.630) and the number of beds in the unit (β 0.008). Larger indirect effects of the NH environment have emerged on the nursing staff, where the amount of interventions performed on a daily basis at the resident level have been increased by ‘Space setting’ (β 3.837), ‘Visual tactile’ (β 1.478), ‘Exit control’ (β 1.037) and ‘Lighting’ (β 0.954) dimensions and prevented by ‘Orientation/cueing’ (− 5.725), ‘Cleanliness’ (− 2.833) ‘Familiarity’ (β − 1.939), ‘Noise’(β − 1.422) and ‘Maintenance’ (β − 0.713) dimensions. The number of interventions performed at the resident level were prevented by the increased Barthel Index (β − 0.668) and increased by the clinical instability (β 0.075).

According to Tarling [34] direct, indirect and total effects have been calculated (Table 3). NH level variables with the largest effects increasing eating-dependence were, in order, ‘Cleanliness’, ‘Orientation/cueing’, ‘Familiarity’ and ‘Maintenance’. NH level variables preventing eating dependence were appropriate ‘Space Setting’ and ‘Lighting’.

**Table 3:** Path analysis findings: direct, indirect and total effects [32]

					TESS-NH dimension total effect^b^		
TESS-NH Dimensions^a^	Direct Effects on EdFED (β)	Indirect effects on Barthel Index (β)	Indirect effects on night restlessness (β)	Indirect effects on interventions at the resident level (β)	+ Barthel Index (β −2.374)	+ Night restlessness (β 0.259)	+ Interventions at the resident level (β −0.524)
Outdoor Access	−1.225						
Privacy	−0.087		0.011			−0.076	
Exit Control	−0.604	− 0.596	− 0.159	− 0.543	− 1.200	−0.763	− 1.147
Maintenance	2.919	0.330		0.374	3.249		3.293
Cleanliness	6.574			1.484			8.058
Safety	−3.055						
Lighting	−2.848	−0.584		−0.500	−3.432		−3.348
Visual Tactile	1.459			−0.774			0.685
Noise	0.361	−0.157		0.745	0.204		1.106
Space Setting	−4.446	−1.059	−0.222	−2.011	−5.505	−4.668	−6.457
Familiarity	2.326	0.325		1.016	2.651		3.342
Orientation/cueing	0.533	2.381	0.422	3.000	2.914	0.955	3.533

^a^Unit autonomy dimension was not entered in the model given it was a constant across the NH units

^b^Direct effect on EdFED + indirect effects

*EdFED* Edinburgh Feeding Evaluation in Dementia scale, *TESS-NH* Therapeutic Environment Screening Survey for Nursing Home

## Discussion

### Individual, nursing care and NH’s variables

More than 35% of residents required from partial to complete help in eating confirming that eating dependence is an increased care need in NHs [4, 38, 39].

The residents’ profile was similar to that reported in previous studies as highly functionally and cognitively impaired, and around four out of ten with a depressed mood [40]. Residents were moderately clinically stable as previously documented [41] and have shown less than one episode/week of behavioural problems such as night restlessness, verbal and physical aggressiveness, suggesting that there was a low incidence of episodes of resistance to cooperate in care [42]. The majority of them were in a close relationship with relatives, in line with previous studies [34].

At the nursing care level, interventions routinely delivered to promote eating performances in the dining environment were on average four, with a great variability across NHs (from two to six/NH). At the residents’ level, on average of more than eight interventions (from one to ten/NH) were performed. Staff attitudes, evidence-based knowledge, beliefs [43], tacit knowledge [44] regarding what is effective and not with each resident, as well as the culture and policies of the NH regarding mealtime [6] can explain these variations.

At the NH level, a moderate pleasant, positive and functional evaluation was evidenced, higher when compared to previous studies where an average of 5.75 has been reported [26]. Specifically, in some dimensions (‘Unit Autonomy’, ‘Exit control’, ‘Maintenance’, ‘Cleanliness’, ‘Safety’, ‘Lighting’, ‘Visual/tactile’, ‘Space setting’ and ‘Orientation/cueing’) the average scores were higher as compared to those documented previously [26, 45]. In the remaining dimensions (‘Outdoor access’, ‘Privacy’, ‘Noise’ and ‘Familiarity’) the average scores were lower as compared to previous studies [26, 45] thus suggesting areas of improvements.

According to the Intra Class Correlations findings, the proportion of the total variability in the outcome variable was limited but higher at the NH units’ level, suggesting that some factors embodied in the micro environment where residents live affect the eating dependence as documented by the following path analysis.

### Path analysis findings

The analysis explained the 57.7% variance in the eating dependence: the cognitive decline contributed a modest amount to eating dependence; while the greatest contribution was functional dependence, as measured by the Barthel Index. This confirms that the decline of ADLs associated with cognitive decline is progressive and ends with eating dependence [46]. Being female and having night restlessness also contributed to the extent of dependence in feeding. The role of sex can be explained by the older age at which they joint the NH [47] which can imply also a lack of family support [48]. Night restlessness can be an indicator of psychological symptoms of cognitive decline, as well as the consequence of inactivity, that can lead to both an increased day time tiredness, difficulty engaging in activities and increased risk of sleep medication (e.g. benzodiazepine administration) due to staff burden [49].

According to the findings, residents who ate in the dining room close to or surrounded by two residents, rather than eating alone in the bedroom, were more likely to eat independently. The presence of family was also associated with eating independently. The social meaning of meals has already been recognised [16, 30, 38]: sitting surrounded by others can give residents the opportunity to mirror some behaviour [50] while significant others can offer a personalised support thus promoting high quality of interaction during mealtimes [51].

Notwithstanding the effect of individual variables, the NH level variables had a larger direct impact on self-feeding dependence. Less supportive environments have already been documented as significantly associated with eating excess disabilities [6]. We found that cleaner and maintained NH units were associated with an increased dependence in eating. This is possibly because a higher attention to these elements can prevent the degree of freedom to residents (e.g., to eat with fingers, to spill out) and the attitude of the staff to totally compensate his/her difficulties.

Environmental familiarity has emerged as also being associated with increased eating dependence as already documented by Keller et al. [38], possibly because familiar, non-institutional furnishings; however, fully offering a familiar environment can be really challenging in a context of safety measures (e.g. automated beds) and where residents from different cultures co-exist (e.g., the need to share a bedroom). Besides, the ‘sense of home’ is multifactorial, not only including the building and interior design but also familiar food and drinks [52]. Although to a lesser extent, the ‘Visual/tactile stimulation’ dimension has emerged as also associated with an increased eating dependence. Possible explanations include excessive distracting stimuli that should be further studied in underlying mechanisms.

On the other side, allowing space for residents, a safe setting, appropriately lit, and open to the external environment were associated with a reduced eating dependence. Perhaps this is because all of these factors affect resident’s engagement in activity [52, 53] thereby promoting independence. Light, noise, and temperature levels have been already documented as reducing self-feeding abilities [27].

The majority of the above-mentioned environmental factors have been reported to have a moderate indirect effect on the degree of functional dependence and on night restlessness, while higher indirect effects on interventions performed by nursing staff, in some dimensions preventing (‘Orientation/cueing’, ‘Cleanliness’, and ‘Familiarity’) while in others increasing their occurrence (‘Space setting’ and ‘Visual tactile’). This seems to confirm that environmental factors at the unit level can have both direct effects and a mediator effects by explaining the 81.1% variance in the nursing care interventions at the resident level. These findings can be interpreted in two ways: on one side the increased quality of some dimensions of the unit environment (e.g., high orientation, cueing, familiarity) can prevent specific stimulation of the resident during mealtime because the environment is mistakenly perceived as a substitute for individualised care. Alternatively, the high maintenance and cleanliness can discourage attempts of the staff to stimulate to eat alone, with a focus on ensuring the highest level of cleanliness and order as expected by the NH. In this light, not only the dining atmosphere as already documented [27, 54] but also the entire environmental factors at the unit level [55] can affect the eating performance due to its intersection both with eating dependence and the care delivered by the staff.

Differently, the number of interventions performed by nurses at the dining room level (e.g. reducing noises, distractions), have reported no direct effects on eating dependence. The variance arisen in these interventions was mainly explained by the quality of the unit environment as measured by the TESS-NH tool. This suggests that the overall quality of the unit environment affects the number of interventions performed at the dining room, but the negative indirect effects in some cases (e.g., ‘Exit control’, ‘Lighting’) and the positive indirect effects in others (‘Space setting’) requires further investigation.

### Limitations

The outcome variable was measured one time, by observing lunch; residents have been documented to have variations in their eating performance over the day and the time, e.g. with an increased degree of cooperation and the physiological capacity to eat at breakfast [18, 40]. Data on residents were collected from their routine assessments as stored in the database; as a consequence, no data on medications were collected. Data on nursing staff as for example, the staff-to-resident, were not collected given the homogenous care offered in the included NHs; in addition, no data regarding staff attitudes or knowledge regarding how to promote eating independence were collected [7].

Given that no interventions can be considered to date as being a gold standard aimed at maintaining or increasing eating performances in residents living in NH [8], we identified interventions via focus groups and then included them in a checklist. The checklist was then used to document the strategies observed in NHs. Further studies aimed at assessing the check list validity is suggested. Moreover, we evaluated only the number of interventions performed in a set of possible interventions all attempting to stimulate each resident to eat independently according to the available research in the field: the intensity applied to these interventions to each resident has not been measured [28], suggesting that further studies should also consider this aspect and not only the number of interventions performed.

## Conclusions

The study findings suggest that the environment of the NH unit generates both direct and indirect positive and negative effects on eating performance, while the amount of environmental interventions enacted by nurses at the dining level have all reported no significant effects.

Eating dependence is a complex phenomenon requiring multiple interventions. Apart from individual unmodifiable predisposing factors (female gender, cognitive decline), some modifiable factors such as: decreased functional dependence, decreased night restlessness, eating in the dining room with others and the presence of close family members, can all reduce eating dependence. At the nursing care level, the number of interventions performed daily to maintain self-feeding independence can prevent self-feeding dependence. However, the largest direct and indirect effect on self-feeding dependence was the quality of the NH unit environment suggesting that there is a need to consider the whole environment where the resident live and not only that of the dining room. Changes are required not only at the dining room level, but in the entire NH unit that should not be ‘perfect’: increased scores in some dimensions (e.g., cleanliness), emerged as both direct and indirect effects on increased eating feeding dependence. Accordingly, further studies aimed at evaluating the best environment capable of maximising eating performance are recommended also with the intent to provide cumulative evidence and to inform the environment design decisions.

Moreover, in studies testing the effectiveness of interventions at the resident level, the quality of the environment should also be evaluated and documented given its role in mediating the degree of eating dependence.

## Supplementary information

**Additional file 1.** Correlations between explanatory variables (at the individual, nursing care and NH levels) and the outcome variable.

**Additional file 2.** Path Analysis Findings.

## Data Availability

The datasets used and/or analysed during the current study are available from the corresponding author on request.

## Abbreviations

ADL: Activity of Daily Living
BI: Barthel Index
CI: Confidence Intervals
CPS: Cognitive Performance Scale
DRS: Depression Rating Scale
EdFED: Edinburgh Feeding Evaluation in Dementia scale
ICC: Intra Class Correlation
NA: Nursing aides
NH: Nursing home
RN: Registered Nurse
SD: Standard Deviations
Std.all: Completely standardized solution
Std.Err: Standard error
Std.lv: Standardized latent variable coefficient
TESS-NH: Therapeutic Environment Screening Survey for Nursing Home
