# Supplementary material for: A path analysis on the direct and indirect effects of the unit environment on eating dependence among cognitively impaired nursing home residents
Source: BMC Health Serv Res. 2019 Oct 30;19:775. doi: 10.1186/s12913-019-4667-z (PMC6822399; doi:10.1186/s12913-019-4667-z)
Supplement: Supplementary file 1 — Additional file 1. Correlations between explanatory variables (at the individual, nursing care and NH levels) and the outcome variable. [file 12913_2019_4667_MOESM1_ESM.docx]

**Additional file 1.**

Correlations between explanatory variables (at the individual, nursing care and NH levels) and the outcome variable

|  | **1 EdFED** | **2 Age** | **3 Sex** | **4 Barthel Index** | **5 Cognitive Performance Scale** | **6 Depression Rating Scale** | **7 Pain Intensity Scale** | **8 Night restlessness** | **9 Verbal aggressiveness** | **10 Physical aggressiveness** | **11 Clinical Instability Scale** | **12 Close relationship with family** | **13 Eating alone, in the bedroom or in the dining room, near one, two or three residents** | **14 Environmental Interventions** | **15 Resident Interventions** | **16 NH bed sizes** | **17 NH Unit bed sizes** | **19 TESS-NH Outdoor Access** | **20 TESS-NH Privacy** | **21 TESS-NH Exit Control** | **22 TESS-NH Maintenance** | **23 TESS-NH Cleanliness** | **24 TESS-NH Safety** | **25 TESS-NH** Lighting | **26 TESS-NH Visual/Tactile** | **27 TESS-NH Noise** | **28 TESS-NH Space Setting** | **29 TESS-NH Familiarity** | **30 TESS-NH Orientation/cueing** | **31 TESS-NH Total global single item** |
| --- | --- | --- | --- | --- | --- | --- | --- | --- | --- | --- | --- | --- | --- | --- | --- | --- | --- | --- | --- | --- | --- | --- | --- | --- | --- | --- | --- | --- | --- | --- |
| **1 Eating dependence as measured with EdFED** | 1.00 |  |  |  |  |  |  |  |  |  |  |  |  |  |  |  |  |  |  |  |  |  |  |  |  |  |  |  |  |  |
| **Individual level** |  |  |  |  |  |  |  |  |  |  |  |  |  |  |  |  |  |  |  |  |  |  |  |  |  |  |  |  |  |  |
| **2 Age** | .107^**^ | 1.00 |  |  |  |  |  |  |  |  |  |  |  |  |  |  |  |  |  |  |  |  |  |  |  |  |  |  |  |  |
| **3 Sex** | .123^**^ | .315^**^ | 1.00 |  |  |  |  |  |  |  |  |  |  |  |  |  |  |  |  |  |  |  |  |  |  |  |  |  |  |  |
| **4 Barthel Index** | -.396^**^ | -.190^**^ | -.165^**^ | 1.00 |  |  |  |  |  |  |  |  |  |  |  |  |  |  |  |  |  |  |  |  |  |  |  |  |  |  |
| **5 Cognitive Performance Scale** | .450^**^ | .078^*^ | .134^**^ | -.597^**^ | 1.00 |  |  |  |  |  |  |  |  |  |  |  |  |  |  |  |  |  |  |  |  |  |  |  |  |  |
| **6 Depression rating Scale** | -0.02 | -0.01 | .124^**^ | -0.03 | 0.01 | 1.00 |  |  |  |  |  |  |  |  |  |  |  |  |  |  |  |  |  |  |  |  |  |  |  |  |
| **7 Pain Intensity** | -0.04 | .096^**^ | .157^**^ | -.067^*^ | -0.05 | .193^**^ | 1.00 |  |  |  |  |  |  |  |  |  |  |  |  |  |  |  |  |  |  |  |  |  |  |  |
| **8 Night restlessness** | .150^**^ | 0.06 | 0.03 | -.170^**^ | .265^**^ | .246^**^ | 0.00 | 1.00 |  |  |  |  |  |  |  |  |  |  |  |  |  |  |  |  |  |  |  |  |  |  |
| **9 Verbal aggressiveness** | 0.00 | -0.06 | -.089^**^ | -0.06 | .078^*^ | .329^**^ | 0.01 | .280^**^ | 1.00 |  |  |  |  |  |  |  |  |  |  |  |  |  |  |  |  |  |  |  |  |  |
| **10 Physical aggressiveness** | .107^**^ | 0.03 | -0.05 | -.091^**^ | .178^**^ | .097^**^ | -0.04 | .231^**^ | .484^**^ | 1.00 |  |  |  |  |  |  |  |  |  |  |  |  |  |  |  |  |  |  |  |  |
| **11 Clinical Instability Scale** | 0.05 | 0.03 | -0.01 | -.229^**^ | .136^**^ | .101^**^ | .110^**^ | .122^**^ | 0.06 | .073^*^ | 1.00 |  |  |  |  |  |  |  |  |  |  |  |  |  |  |  |  |  |  |  |
| **12 Close relationship with family** | -.222^**^ | .093^**^ | .066^*^ | .143^**^ | -.351^**^ | .126^**^ | .114^**^ | -.074^*^ | -0.03 | -.135^**^ | 0.02 | 1.00 |  |  |  |  |  |  |  |  |  |  |  |  |  |  |  |  |  |  |
| **13 Eating alone, in the bedroom or in the dining room, near one, two or three residents** | -.272^**^ | -0.01 | -0.01 | .248^**^ | -.244^**^ | -0.03 | -0.04 | -0.04 | 0.00 | -0.04 | -.110^**^ | .122^**^ | 1.00 |  |  |  |  |  |  |  |  |  |  |  |  |  |  |  |  |  |
| **Nursing Care level** |  |  |  |  |  |  |  |  |  |  |  |  |  |  |  |  |  |  |  |  |  |  |  |  |  |  |  |  |  |  |
| **14 Environmental interventions** | -0.03 | .110^**^ | .100^**^ | .174^**^ | -.067^*^ | .113^**^ | .190^**^ | 0.02 | .098^**^ | 0.02 | -.099^**^ | .064^*^ | .107^**^ | 1.00 |  |  |  |  |  |  |  |  |  |  |  |  |  |  |  |  |
| **15 Resident interventions** | 0.03 | -0.02 | .102^**^ | -.296^**^ | .233^**^ | 0.03 | .069^*^ | 0.05 | 0.00 | 0.00 | .173^**^ | -.077^*^ | -.149^**^ | -.333^**^ | 1.00 |  |  |  |  |  |  |  |  |  |  |  |  |  |  |  |
| **NH level** |  |  |  |  |  |  |  |  |  |  |  |  |  |  |  |  |  |  |  |  |  |  |  |  |  |  |  |  |  |  |
| **16 NH bed size** | -0.03 | -0.02 | 0.00 | -.220^**^ | .078^*^ | -.071^*^ | -0.01 | 0.05 | 0.00 | -0.01 | .162^**^ | 0.01 | 0.01 | -.247^**^ | .530^**^ | 1.00 |  |  |  |  |  |  |  |  |  |  |  |  |  |  |
| **17 NH Unit bed size** | 0.01 | 0.04 | -0.06 | -.191^**^ | 0.03 | 0.02 | -0.05 | .119^**^ | .068^*^ | 0.06 | .183^**^ | .067^*^ | .063^*^ | 0.01 | .145^**^ | .725^**^ | 1.00 |  |  |  |  |  |  |  |  |  |  |  |  |  |
| **19 TESS-NH Outdoor Access** | -.162^**^ | .077^*^ | 0.01 | -0.01 | 0.02 | .108^**^ | 0.01 | .073^*^ | .111^**^ | 0.04 | .203^**^ | 0.05 | 0.04 | .181^**^ | -.073^*^ | .263^**^ | .460^**^ | 1.00 |  |  |  |  |  |  |  |  |  |  |  |  |
| **20 TESS-NH Privacy** | 0.02 | .118^**^ | .123^**^ | .098^**^ | 0.02 | .094^**^ | .159^**^ | -0.01 | .073^*^ | -0.01 | -.091^**^ | 0.03 | -.079^*^ | .454^**^ | .100^**^ | -.277^**^ | -.284^**^ | -.239^**^ | 1.00 |  |  |  |  |  |  |  |  |  |  |  |
| **21 TESS-NH Exit Control** | 0.03 | 0.03 | -.101^**^ | -.082^**^ | 0.00 | -.211^**^ | -.097^**^ | -.074^*^ | -0.02 | -0.03 | .133^**^ | -0.04 | -0.03 | -.266^**^ | .127^**^ | .319^**^ | .199^**^ | .199^**^ | -.232^**^ | 1.00 |  |  |  |  |  |  |  |  |  |  |
| **22 TESS-NH Maintenance** | -0.03 | .064^*^ | 0.04 | -0.02 | 0.03 | -0.01 | -0.02 | -0.05 | 0.03 | -0.02 | .077^*^ | -0.06 | .115^**^ | .069^*^ | .219^**^ | .103^**^ | 0.02 | .347^**^ | 0.00 | .421^**^ | 1.00 |  |  |  |  |  |  |  |  |  |
| **23 TESS-NH Cleanliness** | 0.05 | 0.02 | -.083^**^ | 0.06 | 0.00 | -0.05 | -0.04 | -0.05 | 0.03 | 0.01 | .125^**^ | -0.02 | 0.03 | .127^**^ | -0.06 | 0.01 | .160^**^ | .191^**^ | .226^**^ | .387^**^ | .468^**^ | 1.00 |  |  |  |  |  |  |  |  |
| **24 TESS-NH Safety** | 0.00 | -0.02 | -.104^**^ | -0.04 | 0.01 | -0.02 | -.102^**^ | 0.00 | 0.03 | 0.01 | .154^**^ | -0.03 | 0.05 | -.078^*^ | 0.06 | .258^**^ | .331^**^ | .393^**^ | -.196^**^ | .491^**^ | .635^**^ | .753^**^ | 1.00 |  |  |  |  |  |  |  |
| **25 TESS-NH Lighting** | -.113^**^ | -.102^**^ | -.076^*^ | .108^**^ | -0.01 | 0.05 | -.068^*^ | 0.01 | -0.03 | -0.01 | .097^**^ | -.067^*^ | -0.01 | -.082^**^ | -0.05 | 0.01 | -0.02 | .321^**^ | -.200^**^ | -0.06 | .345^**^ | .473^**^ | .600^**^ | 1.00 |  |  |  |  |  |  |
| **26 TESS-NH Visual/Tactile** | 0.02 | 0.05 | 0.05 | -0.06 | .124^**^ | .064^*^ | -0.01 | 0.01 | 0.03 | 0.01 | .227^**^ | -0.04 | -.125^**^ | -.065^*^ | .356^**^ | -.159^**^ | -.191^**^ | .271^**^ | .196^**^ | .224^**^ | .550^**^ | .434^**^ | .492^**^ | .371^**^ | 1.00 |  |  |  |  |  |
| **27 TESS-NH Noise** | -.127^**^ | 0.06 | 0.00 | .207^**^ | -.082^**^ | .135^**^ | .094^**^ | 0.05 | .083^**^ | 0.06 | 0.00 | 0.06 | .084^**^ | .676^**^ | -.458^**^ | -.122^**^ | .199^**^ | .563^**^ | .094^**^ | -.211^**^ | .120^**^ | .170^**^ | .139^**^ | .318^**^ | -0.06 | 1.00 |  |  |  |  |
| **28 TESS-NH Space Setting** | .400^**^ | .154^**^ | .131^**^ | -0.02 | .130^**^ | 0.03 | .079^*^ | 0.02 | 0.04 | 0.01 | .116^**^ | -0.05 | -.089^**^ | .460^**^ | 0.04 | -.224^**^ | -.128^**^ | .276^**^ | .398^**^ | .249^**^ | .513^**^ | .473^**^ | .318^**^ | .105^**^ | .585^**^ | .268^**^ | 1.00 |  |  |  |
| **29 TESS-NH Familiarity** | 0.04 | .150^**^ | .100^**^ | .113^**^ | -.094^**^ | 0.02 | .117^**^ | -.084^**^ | 0.03 | -0.03 | -0.01 | .122^**^ | -.065^*^ | .493^**^ | -.228^**^ | -.439^**^ | -.234^**^ | .212^**^ | .289^**^ | .116^**^ | .166^**^ | .269^**^ | .140^**^ | -0.03 | .442^**^ | .247^**^ | .624^**^ | 1.00 |  |  |
| **30 TESS-NH Orientation/cueing** | -0.02 | 0.03 | 0.02 | -0.03 | .126^**^ | 0.01 | -0.01 | 0.00 | 0.01 | 0.01 | .223^**^ | -.113^**^ | -.102^**^ | 0.05 | .112^**^ | -.401^**^ | -.317^**^ | .240^**^ | .079^*^ | .351^**^ | .423^**^ | .314^**^ | .257^**^ | .241^**^ | .716^**^ | 0.01 | .566^**^ | .382^**^ | 1.00 |  |
| **31 TESS-NH Total global single item** | -.085^**^ | .103^**^ | .092^**^ | .070^*^ | 0.05 | 0.04 | .103^**^ | -0.03 | .065^*^ | -0.02 | .151^**^ | -0.02 | 0.00 | .489^**^ | .143^**^ | -.101^**^ | -.085^**^ | .455^**^ | .417^**^ | .287^**^ | .678^**^ | .520^**^ | .403^**^ | .233^**^ | .653^**^ | .376^**^ | .795^**^ | .487^**^ | .665^**^ | 1.00 |

*p-value <.05 (two tailes); **p-value <.01 (two tailes)

*EdFED* Edinburgh Feeding Evaluation in Dementia scale, *NH* Nursing Homes, *TESS-NH* Therapeutic Environment Screening Survey for Nursing Home.

The variable n. 18 ‘TESS-NH Unit Autonomy’ was removed because the score was constant across NHs.
