# Supplementary material for: A path analysis on the direct and indirect effects of the unit environment on eating dependence among cognitively impaired nursing home residents
Source: BMC Health Serv Res. 2019 Oct 30;19:775. doi: 10.1186/s12913-019-4667-z (PMC6822399; doi:10.1186/s12913-019-4667-z)
Supplement: Supplementary file 2 — Additional file 2. Path Analysis Findings. [file 12913_2019_4667_MOESM2_ESM.docx]

**Additional file 2**

Path Analysis Findings

| **Regressions** |  |  |  |  |
| --- | --- | --- | --- | --- |
|  | **Estimate** | **Std.Err** | **z-value** | **P(>\|z\|)** |
| **Outcome: eating dependence (EdFED) ~** | |  |  |  |
| Age | 0.005 | 0.013 | 0.409 | 0.682 |
| Female | 0.700 | 0.275 | 2.541 | 0.011 |
| Barthel Index | -2.374 | 0.531 | -4.471 | <.001 |
| Cognitive Performance Scale | 0.600 | 0.075 | 7.964 | <.001 |
| Depression Rating Scale | -0.194 | 0.156 | -1.246 | 0.213 |
| Pain Intensity | 0.155 | 0.531 | 0.292 | 0.770 |
| Night restlessness | 0.259 | 0.117 | 2.204 | 0.028 |
| Verbal aggressiveness | -0.296 | 0.170 | -1.746 | 0.081 |
| Physical aggressiveness | 0.278 | 0.233 | 1.194 | 0.232 |
| Clinical Instability Score | 0.003 | 0.134 | 0.023 | 0.982 |
| Close relationships with family relatives | -0.854 | 0.265 | -3.225 | 0.001 |
| Dining room, near one resident (on left or right side) | -0.702 | 0.527 | -1.331 | 0.183 |
| Dining room, near two residents (on left or right side) | -1.352 | 0.431 | -3.141 | 0.002 |
| Dining room, near two residents (on left/right and in front) | -1.577 | 0.433 | -3.644 | <.001 |
| Dining room, surrounded by other residents | -1.802 | 0.301 | -5.985 | <.001 |
| Interventions at Residents level | -0.524 | 0.176 | -2.977 | 0.003 |
| Interventions at Environmental level | 0.087 | 0.280 | 0.310 | 0.757 |
| Unit beds | 0.041 | 0.020 | 2.066 | 0.039 |
| NH Units, number | 0.467 | 0.309 | 1.510 | 0.131 |
| TESS-NH Outdoor Access | -1.225 | 0.353 | -3.466 | 0.001 |
| TESS-NH Privacy | -0.087 | 0.076 | -1.150 | 0.250 |
| TESS-NH Exit Control | -0.604 | 1.407 | -0.429 | 0.668 |
| TESS-NH Maintenance | 2.919 | 0.780 | 3.740 | <.001 |
| TESS-NH Cleanliness | 6.574 | 1.664 | 3.951 | <.001 |
| TESS-NH Safety | -3.055 | 1.345 | -2.272 | 0.023 |
| TESS-NH Lighting | -2.848 | 1.378 | -2.067 | 0.039 |
| TESS-NH Visual Tactile | 1.459 | 0.633 | 2.305 | 0.021 |
| TESS-NH Noise | 0.361 | 0.485 | 0.745 | 0.456 |
| TESS-NH Space Setting | -4.446 | 1.779 | -2.498 | 0.012 |
| TESS-NH Familiarity | 2.326 | 0.613 | 3.793 | <.001 |
| TESS-NH Orientation/cueing | 0.533 | 2.551 | 0.209 | 0.834 |
| **Barthel Index ~** |  |  |  |  |
| Unit beds | -0.007 | 0.001 | -7.292 | <.001 |
| NH Units, number | -0.071 | 0.018 | -3.901 | <.001 |
| TESS-NH Outdoor Access | -0.004 | 0.021 | -0.178 | 0.859 |
| TESS-NH Privacy | -0.009 | 0.006 | -1.520 | 0.128 |
| TESS-NH Exit Control | 0.251 | 0.087 | 2.904 | 0.004 |
| TESS-NH Maintenance | -0.139 | 0.059 | -2.352 | 0.019 |
| TESS-NH Cleanliness | 0.065 | 0.123 | 0.531 | 0.595 |
| TESS-NH Safety | -0.103 | 0.100 | -1.035 | 0.300 |
| TESS-NH Lighting | 0.246 | 0.101 | 2.422 | 0.015 |
| TESS-NH Visual Tactile | -0.043 | 0.039 | -1.098 | 0.272 |
| TESS-NH Noise | 0.066 | 0.031 | 2.144 | 0.032 |
| TESS-NH Space Setting | 0.446 | 0.116 | 3.857 | <.001 |
| TESS-NH Familiarity | -0.137 | 0.045 | -3.043 | 0.002 |
| TESS-NH Orientation/cueing | -1.003 | 0.184 | -5.458 | <.001 |
| **Night restlessness ~** |  |  |  |  |
| Unit beds | 0.008 | 0.003 | 2.227 | 0.026 |
| NH Units, number | 0.029 | 0.068 | 0.427 | 0.670 |
| TESS-NH Outdoor Access | 0.069 | 0.080 | 0.860 | 0.390 |
| TESS-NH Privacy | 0.044 | 0.021 | 2.063 | 0.039 |
| TESS-NH Exit Control | -0.614 | 0.324 | -1.896 | 0.058 |
| TESS-NH Maintenance | -0.075 | 0.221 | -0.341 | 0.733 |
| TESS-NH Cleanliness | -0.536 | 0.462 | -1.161 | 0.246 |
| TESS-NH Safety | 0.489 | 0.373 | 1.309 | 0.190 |
| TESS-NH Lighting | -0.532 | 0.380 | -1.399 | 0.162 |
| TESS-NH Visual Tactile | 0.233 | 0.145 | 1.606 | 0.108 |
| TESS-NH Noise | 0.212 | 0.116 | 1.832 | 0.067 |
| TESS-NH Space Setting | -0.856 | 0.434 | -1.974 | 0.048 |
| TESS-NH Familiarity | -0.064 | 0.169 | -0.381 | 0.703 |
| TESS-NH Orientation/cueing | 1.630 | 0.688 | 2.367 | 0.018 |
| **Interventions at Residents levels ~** |  |  |  |  |
| Barthel Index | -0.668 | 0.135 | -4.958 | <.001 |
| Cognitive Performance Scale | 0.029 | 0.019 | 1.525 | 0.127 |
| Depression rating Scale | 0.232 | 0.140 | 1.649 | 0.099 |
| Pain Intensity | 0.064 | 0.042 | 1.535 | 0.125 |
| Night restlessness | 0.005 | 0.031 | 0.174 | 0.862 |
| Verbal aggressiveness | -0.033 | 0.045 | -0.738 | 0.461 |
| Physical aggressiveness | 0.049 | 0.062 | 0.793 | 0.428 |
| Clinical Instability Score | 0.075 | 0.035 | 2.100 | 0.036 |
| TESS-NH Outdoor Access | -0.001 | 0.065 | -0.014 | 0.989 |
| TESS-NH Privacy | 0.005 | 0.019 | 0.244 | 0.807 |
| TESS-NH Exit Control | 1.037 | 0.291 | 3.568 | <.001 |
| TESS-NH Maintenance | -0.713 | 0.196 | -3.643 | <.001 |
| TESS-NH Cleanliness | -2.833 | 0.392 | -7.230 | <.001 |
| TESS-NH Safety | -0.254 | 0.327 | -0.777 | 0.437 |
| TESS-NH Lighting | 0.954 | 0.309 | 3.091 | 0.002 |
| TESS-NH Visual Tactile | 1.478 | 0.135 | 10.986 | <.001 |
| TESS-NH Noise | -1.422 | 0.101 | -14.130 | <.001 |
| TESS-NH Space Setting | 3.837 | 0.328 | 11.679 | <.001 |
| TESS-NH Familiarity | -1.939 | 0.114 | -16.982 | <.001 |
| TESS-NH Orientation/cueing | -5.725 | 0.458 | -12.491 | <.001 |
| **Interventions at Environmental levels ~** | |  |  |  |
| Barthel Index | -0.063 | 0.067 | -0.940 | 0.347 |
| Cognitive Performance Scale | 0.008 | 0.010 | 0.793 | 0.427 |
| Depression Rating Scale | 0.004 | 0.070 | 0.060 | 0.952 |
| Pain Intensity | 0.018 | 0.021 | 0.875 | 0.381 |
| Night restlessness | 0.042 | 0.016 | 2.668 | 0.008 |
| Verbal aggressiveness | 0.031 | 0.022 | 1.374 | 0.170 |
| Physical aggressiveness | 0.006 | 0.031 | 0.187 | 0.852 |
| Clinical Instability Score | -0.021 | 0.018 | -1.174 | 0.240 |
| TESS-NH Outdoor Access | -0.467 | 0.032 | -14.425 | <.001 |
| TESS-NH Privacy | -0.021 | 0.010 | -2.171 | 0.030 |
| TESS-NH Exit Control | -2.923 | 0.145 | -20.131 | <.001 |
| TESS-NH Maintenance | -0.720 | 0.098 | -7.364 | <.001 |
| TESS-NH Cleanliness | -0.897 | 0.196 | -4.586 | <.001 |
| TESS-NH Safety | 1.928 | 0.163 | 11.807 | <.001 |
| TESS-NH Lighting | -1.888 | 0.154 | -12.248 | <.001 |
| TESS-NH Visual Tactile | -1.422 | 0.067 | -21.167 | <.001 |
| TESS-NH Noise | 0.917 | 0.050 | 18.252 | <.001 |
| TESS-NH Space Setting | 4.126 | 0.164 | 25.157 | <.001 |
| TESS-NH Familiarity | 0.117 | 0.057 | 2.047 | 0.041 |
| TESS-NH Orientation/cueing | -0.027 | 0.229 | -0.118 | 0.906 |
| **Outdoor Access ~** |  |  |  |  |
| Unit beds | 0.014 | 0.001 | 11.832 | <.001 |
| NH Units, number | -0.159 | 0.025 | -6.397 | <.001 |
| **Privacy ~** |  |  |  |  |
| Unit beds | -0.042 | 0.005 | -8.768 | <.001 |
| NH Units, number | -0.233 | 0.104 | -2.234 | 0.026 |
| **Exit Control ~** |  |  |  |  |
| Unit beds | 0.002 | <.001 | 5.276 | <.001 |
| NH Units, number | -0.004 | 0.006 | -0.626 | 0.531 |
| **Maintenance ~** |  |  |  |  |
| Unit beds | <.001 | <.001 | 0.882 | 0.378 |
| NH Units, number | -0.001 | 0.011 | -0.074 | 0.941 |
| **Cleanliness ~** |  |  |  |  |
| Unit beds | <.001 | <.001 | -0.210 | 0.834 |
| NH Units, number | -0.060 | 0.005 | -10.970 | <.001 |
| **Safety ~** |  |  |  |  |
| Unit beds | 0.003 | <.001 | 6.947 | <.001 |
| NH Units, number | -0.051 | 0.008 | -6.145 | <.001 |
| **Lighting ~** |  |  |  |  |
| Unit beds | -0.001 | <.001 | -2.794 | 0.005 |
| NH Units, number | -0.030 | 0.006 | -4.776 | <.001 |
| **Visual Tactile ~** |  |  |  |  |
| Unit beds | -0.009 | 0.001 | -10.828 | <.001 |
| NH Units, number | -0.193 | 0.017 | -11.206 | <.001 |
| **Noise ~** |  |  |  |  |
| Unit beds | 0.002 | 0.001 | 2.509 | 0.012 |
| NH Units, number | -0.147 | 0.019 | -7.819 | <.001 |
| **Space Setting ~** |  |  |  |  |
| Unit beds | -0.004 | 0.001 | -8.324 | <.001 |
| NH Units, number | -0.118 | 0.011 | -10.302 | <.001 |
| **Familiarity ~** |  |  |  |  |
| Unit beds | -0.011 | 0.001 | -16.966 | <.001 |
| NH Units, number | -0.276 | 0.014 | -19.284 | <.001 |
| **Orientation/cueing ~** |  |  |  |  |
| Unit beds | -0.003 | <.001 | -19.065 | <.001 |
| NH Units, number | -0.071 | 0.004 | -17.799 | <.001 |
|  |  |  |  |  |
| **Intercepts:** | **Estimate** | **Std.Err** | **z-value** | **P(>\|z\|)** |
| Eating dependence (EdFED total) | 2.448 | 2.934 | 0.834 | 0.404 |
| Barthel Index | -0.125 | 0.169 | -0.741 | 0.459 |
| Night restlessness | 1.786 | 0.633 | 2.823 | 0.005 |
| Interventions at Residents level | 9.947 | 0.509 | 19.526 | <.001 |
| Interventions at Environmental level | 5.474 | 0.254 | 21.520 | <.001 |
| TESS-NH Outdoor Access | 2.744 | 0.071 | 38.481 | <.001 |
| TESS-NH Privacy | 2.332 | 0.298 | 7.820 | <.001 |
| TESS-NH Exit Control | 0.939 | 0.018 | 52.085 | <.001 |
| TESS-NH Maintenance | 1.743 | 0.030 | 57.272 | <.001 |
| TESS-NH Cleanliness | 2.086 | 0.016 | 133.203 | <.001 |
| TESS-NH Safety | 2.020 | 0.024 | 84.937 | <.001 |
| TESS-NH Lighting | 1.941 | 0.018 | 109.466 | <.001 |
| TESS-NH Visual Tactile | 2.980 | 0.049 | 60.619 | <.001 |
| TESS-NH Noise | 2.210 | 0.054 | 41.002 | <.001 |
| TESS-NH Space Setting | 1.911 | 0.033 | 58.134 | <.001 |
| TESS-NH Familiarity | 1.593 | 0.041 | 38.919 | <.001 |
| TESS-NH Orientation/cueing | 0.581 | 0.011 | 50.700 | <.001 |
|  |  |  |  |  |
| **Variances:** | **Estimate** | **Std.Err** | **Std.lv** | **Std.all** |
| Eating dependence (EdFED total) | 11.091 | 0.499 | 11.091 | 0.423 |
| Barthel Index | 0.069 | 0.003 | 0.069 | 0.616 |
| Night restlessness | 0.975 | 0.044 | 0.975 | 0.837 |
| Interventions at Residents level | 0.809 | 0.036 | 0.809 | 0.189 |
| Interventions at Environmental level | 0.202 | 0.009 | 0.202 | 0.065 |
| TESS-NH Outdoor Access | 0.436 | 0.020 | 0.436 | 0.749 |
| TESS-NH Privacy | 7.616 | 0.343 | 7.616 | 0.923 |
| TESS-NH Exit Control | 0.028 | 0.001 | 0.028 | 0.960 |
| TESS-NH Maintenance | 0.079 | 0.004 | 0.079 | 0.999 |
| TESS-NH Cleanliness | 0.021 | 0.001 | 0.021 | 0.865 |
| .TESS-NH Safety | 0.048 | 0.002 | 0.048 | 0.856 |
| TESS-NH Lighting | 0.027 | 0.001 | 0.027 | 0.977 |
| TESS-NH Visual Tactile | 0.207 | 0.009 | 0.207 | 0.858 |
| TESS-NH Noise | 0.249 | 0.011 | 0.249 | 0.897 |
| TESS-NH Space Setting | 0.093 | 0.004 | 0.093 | 0.891 |
| TESS-NH Familiarity | 0.143 | 0.006 | 0.143 | 0.688 |
| TESS-NH Orientation/cueing | 0.011 | 0.001 | 0.011 | 0.683 |
|  |  |  |  |  |
| **Model fit measure** |  |  |  |  |
| **R*^2^*** | **Estimate** |  |  |  |
| Eating dependence (EdFED total) | 0.577 |  |  |  |
| Barthel Index | 0.384 |  |  |  |
| Night restlessness | 0.163 |  |  |  |
| Interventions at Residents levels | 0.811 |  |  |  |
| Interventions at Environmental levels | 0.935 |  |  |  |
| TESS-NH Outdoor Access | 0.251 |  |  |  |
| TESS-NH Privacy | 0.077 |  |  |  |
| TESS-NH Exit Control | 0.040 |  |  |  |
| TESS-NH Maintenance | 0.001 |  |  |  |
| TESS-NH Cleanliness | 0.135 |  |  |  |
| TESS-NH Safety | 0.144 |  |  |  |
| TESS-NH Lighting | 0.023 |  |  |  |
| TESS-NH Visual Tactile | 0.142 |  |  |  |
| TESS-NH Noise | 0.103 |  |  |  |
| TESS-NH Space Setting | 0.109 |  |  |  |
| TESS-NH Familiarity | 0.312 |  |  |  |
| TESS-NH Orientation/cueing | 0.317 |  |  |  |

*EdFED* Edinburgh Feeding Evaluation in Dementia scale, *NH* nursing home*, Std.Err* standard error, *Std.lv* Standardized latent variable coefficient, *Std.all* completely standardized solution
